# Are Unhygienic Practices During the Menstrual, Partum and Postpartum Periods Risk Factors for Secondary Infertility?

**Published:** 2007-06

**Authors:** Tazeen Saeed Ali, Neelofar Sami, Ali Khan Khuwaja

**Affiliations:** 1 Department of Community Health Sciences, School of Nursing; 2 Department of Community Health Sciences; 3 Departments of Community Health Sciences and Family Medicine, Aga Khan University, Karachi, Pakistan

**Keywords:** Case-control studies, Hygiene, Infertility, Secondary, Risk factors, Pakistan

## Abstract

This study was aimed at identifying practices during the menstrual, partum and postpartum periods posing possible risk factors contributing towards secondary infertility in women of a selected population in Karachi, Pakistan. A matched case-control study was conducted from April 2003 to March 2004. Four hundred cases were selected from five infertility clinics affiliated with tertiary-care hospitals, and 400 age-matched controls were recruited from the neighbourhood of each case. After taking written consents, trained interviewers conducted interviews using a pretested structured questionnaire. Factors found to be independently associated with secondary infertility were: previous delivery at an unclean place (adjusted odds ratio [AOR]=1.7, 95% confidence interval [CI] 1.1-2.6), delivery by a birth attendant without washing hands with soap (AOR=4.2, 95% CI 2.36-7.47), use of unclean material for absorption of lochia (AOR=3.1, 95% CI 1.5-6.5), non-washing of perineal area after urination/defaecation (AOR=7.1, 95% CI 1.4-35.7), and insertion of home-made vaginal medications (AOR=2.5, 95% CI 1.3-4.7). Since these factors are preventable/modifiable to a great extent, public-health interventions are, thus, recommended to address these risk factors at various levels.

## INTRODUCTION

Secondary infertility refers to couples who are unable to conceive after one year of unprotected intercourse after a previous pregnancy in the reproductive age-group. Globally, approximately, 10-15% of couples are infertile, and secondary infertility outnumbers primary infertility (1). The prevalence of infertility in Pakistan is 21.9%: primary infertility is 3.9%, and secondary infertility is 18.0% (2,3). A number of causes have been identified for infertility from different parts of the world. The most common causes reported are reproductive tract infections (RTIs) (4–6). Upper RTIs in women result in serious consequences, such as pelvic inflammatory disease and adhesions, resulting in infertility (7,8). RTIs are caused by sexually transmitted infections (STIs), endogenous infections resulting from overgrowth of organisms normally existing in the reproductive tract, or through iatrogenic infection as a result of bacteria being introduced into the normally-sterile environment of the upper reproductive tract (9) as in the case of unhygienic practices in the menstruation, natal and postnatal periods.

Unhygienic practices during menstruation have been reported, including use of unhygienic material for the absorption of blood and altered bathing practices. In a study, 26% of women reported to bath less frequently and used unhygienic material to absorb the menstrual flow (10). A study in India reported that only 5.2% of females used sanitary pads, and 77% used old pieces of cloth, while others used a combination of the two (11). Another study from Bangladesh showed that 80% of females re-used the same cloth for absorption of blood, and 42% of them dried the cloth under sun before re-use (12). The study further reported that, at times, material was washed at hidden places but not sun-dried. The material dried in hidden places often remains damp, which may give rise to microbial growth and insect larvae (12). Such practices might result in foul-smelling vaginal discharge (11,13).

Similarly, unhygienic practices during the postpartum period might result in RTIs. Various studies have been conducted in the South Asian population, of which some studies from Pakistan also reported insertion of herbal medicine inside the vagina or uterus during the postpartum period and washing of the perineum with unsafe material, thereby augmenting transmission of micro-organisms to the upper reproductive tract leading to pelvic inflammatory disease culminating in adhesions and infertility (12,14).

Deliveries conducted in hospitals and clinics and those conducted by trained personnel play an important role in the prevention of vaginal infections (15). In Pakistan, 90% of deliveries are conducted at home, and 76% of these are attended by untrained traditional birth attendants (TBAs) (16), which could be added up as a risk factor for vaginal infections and its consequences. However, to the best of our knowledge, in Pakistan, none of the studies have reported such practices to be associated with secondary infertility. Thus, we embarked on this study aimed at identifying the practices during the menstrual, partum and postpartum periods posing risk factors for secondary infertility in women living in Karachi.

## MATERIALS AND METHODS

This case-control study was conducted from April 2003 to March 2004 in Karachi, which is the largest city and economic hub of Pakistan. Cases were defined as currently-married women, aged 15-35 years, with at least one previous conception, irrespective of its outcome and attending one of the five selected infertility clinics. To get maximum diversity of social, cultural and economical status, cases were recruited from five selected infertility clinics in Karachi affiliated with tertiary-care hospitals representing both private and public-health sectors. Age-matched residents within a span of 50 households of the same neighbourhood, to which cases belonged, were enrolled in the control arm. All the controls had at least two livebirths with minimum two years’ duration of stay in that area, similar to the corresponding participants in the case arm. After enrolling the woman as a case from one of the selected infertility clinics, within one week, a data-collector visited the neighbourhood of that particular case and knocked on the door of a house less than 50 households away from case to ask for any married women living in the house and if any married women contacted fulfilled the criteria as a study control they were asked to participate in the study. In the case of refusal or if the woman in that particular household did not meet the inclusion criteria as a control, the data-collector moved to the next household.

We used the following operational definitions of variables of interest:

Secondary infertility: Condition of a couple who has conceived once, irrespective of the outcome of that pregnancy, and is trying to conceive again at least for the last one year.

Clean place of delivery: Hospitals or maternity homes having the facility of a labour room were defined as clean places. All other places, such as home and clinics (healthcare facilities) without having facility of a labour room were considered unclean.

Birth attendant's hand-washing before delivery: The practice of washing hands with soap before delivery by birth attendants, i.e. “hands washed: Yes”, was defined as safe practices, while all others “hands washed: No” were defined as hands not washed, hands washed with water only, and do not remember or do not know.

Trained birth attendant: Physicians, midwives, lady health visitors, and nurses were defined as trained birth attendants, while all other birth attendants (lady health worker, dai, relative, neighbour, self) were defined as untrained.

Clean material to absorb menstrual/locha flow: Use of sanitary pads, new clothes, and washed clothes dried in sunlight for absorbing menstrual blood and lochia during the postpartum period were considered clean material. All other categories of material used (cotton, unwashed rags, washed rags dried inside room) were cate-gorized as unclean material.

Unwashed perineal area: In the postpartum period, cleaning of the perineum with water after urination and defaecation was defined as a safe practice. All other practices, such as non-washing or cleaning of the perineum without water, for example, with the help of cloth, were defined as an unhygienic practice.

Vaginal insertion of home-made medication: Intravaginal insertion of home-made or daimade medication during the postpartum period was defined as an unhygienic practice.

The Ethical Research Committee of Aga Khan University, Karachi, gave ethical approval for conducting the study. Consent for interviewing the cases was taken from the administrative authorities of selected infertility clinics and from the recruited infertile women. Finally, verbal consent was taken from each case and control.

By assuming the proportion of women exposed to an unclean birth-place among controls ranging from 10% to 60% and to detect an OR of 2 at 5% level of significance and power of at least 80% and based on equal sample sizes, we needed at least 360 cases and 360 controls. Considering the dropout rate of 10%, we collected required information from 400 cases and 400 controls.

Trained interviewers collected data using the structured and pretested sets of questionnaire.

Along with variables for demographic and socioeconomic characteristics, questions regarding practices during the menstrual, partum and postpartum periods were also included.

Data were double-entered and validated before final analysis. The SPSS software (version 14) was used for analyzing the data. Frequencies were calculated for socioeconomic variables for each of the study groups. Univariate analysis was done to assess the relationship between secondary infertility and menstrual, partum and postpartum practices using crude ORs with 95% confidence intervals. Multivariate logistic regression was carried out to evaluate the combined effect of multiple factors associated with secondary infertility among cases. Results are presented in terms of adjusted odds ratios (AORs), which express the magnitude of the effect of each category on the outcome, relative to the reference category.

## RESULTS

Personal characteristics of the study participants are shown in Table 1. The mean age of the cases and controls was 28.9±4.3 years and 29.0±4.3 years respectively, and the mean age of the cases and controls at marriage was 18.8±3.3 years and 19.2±3.6 years respectively. The mean duration of current marriage was 10.0±4.8 years for the cases and 10.5±5.0 years for the controls. Fifty-two percent of the cases were literate compared to 67% of the controls. Only 12.5% of the cases were employed, while 24% of the controls were employed.

**Table 1:** Personal characteristics of study subjects

Characteristics	Cases (n=400) No. (%)	Controls (n=400) No. (%)
Mean age (years) of respondents	28.9±4.3 SD	29.0±4.3 SD
Mean age (years) at marriage	18.8±3.3 SD	19.2±3.6 SD
Mean duration (years) of current marriage	10.0±4.8	10.5±5.0 SD
Literacy of women	207 (51.8)	267 (66.8)
Literacy of husbands	284 (71.0)	298 (74.5)
Occupation of respondent		
Housewife	350 (87.5)	304 (76.0)
Employed	50 (12.5)	96 (24.0)

SD=Standard deviation

Reported practices during the menstrual, partum and postpartum periods by the study participants and their association with secondary infertility are described in Table 2. The factors associated with secondary infertility in univariate analysis were unclean place of delivery, conducting delivery by a birth attendant without washing hands with soap, use of unclean sheet for delivery, conducting delivery by an untrained birth attendant, use of unhygienic gloves during delivery, use of unclean instruments at the time of delivery, use of unclean material to absorb the menstrual flow, use of unclean material for the absorption of lochia, avoiding bathing during the postpartum period, not washing perineal area after urination or defaecation during the last postpartum period, and intravaginal home-made medication.

**Table 2:** Reported practices during the menstrual, partum and postpartum periods and their association with secondary infertility

Variable	Cases		Controls		Odds ratio (CI 95%)	Adjusted odds ratio (CI 95%)
	No.	%	No.	%		
Unclean place of delivery	218	54.5	152	38.0	2.1 (1.5-2.8)	1.7 (1.1-2.6)
Birth attendant not washed hands with soap before delivery	164	41.0	69	17.3	4.3 (3.0-6.4)	4.2 (2.4-7.4)
No use of clean sheets during delivery	128	32.0	35	8.8	5.0 (3.2-7.9)	NS
No use of gloves at the time of delivery	156	39.0	87	21.8	2.6 (1.8-3.6)	NS
No use of clean instruments	186	46.5	150	37.5	1.5 (1.1-2.0)	NS
Delivery by untrained birth attendant	131	32.8	95	23.8	1.6 (1.2-2.3)	NS
No use of clean material to absorb menstrual flow	210	52.5	137	34.3	2.8 (1.9-4.0)	NS
No use of clean material to absorb lochia	364	91.0	336	84.0	2.0 (1.3-3.1)	3.1 (1.5-6.5)
No bath during the postpartum period	141	35.3	159	39.8	2.8 (1.7-4.6)	NS
No washing of perineal area after defaecation and urination	395	98.8	360	90.0	8.0 (3.2-20.3)	7.1 (1.4-35.7)
Vaginal insertion of home-made medication	84	21.0	41	10.3	2.7 (1.7-4.3)	2.5 (1.3-4.7)

CI=Confidence interval; NS=Not significant

In the final multivariate analysis, five factors were independently associated with secondary infertility. These were: unclean place of delivery (AOR=1.7, 95% confidence interval [CI] 1.1-2.6), delivery by the birth attendant without washing hands with soap (AOR=4.2, 95% CI 2.4-7.4), use of unclean material for the absorption of lochia (AOR=3.1, 95% CI 1.5-6.5), not washing perineal area after urination and defaecation during the last postpartum period (AOR=7.1, 95% CI 1.4-35.7), and use of intravaginal home-made medications (AOR=2.5, 95% CI 1.3-4.7) (Table 2).

## DISCUSSION

Infertility—whether primary or secondary—is an experience that strikes at the very core of a woman's life. Inability to reproduce the desired number of children results in catastrophe that negatively impacts a woman's relationship (17–19) not only with the husband but also with other family members leading to destabilization of her social status (20). These entire physical and psychological traumas can be prevented by simple hygienic interventions at the menstrual, partum and postpartum periods. This study was conducted to identify the risk factors of secondary infertility during the menstruation, partum and postpartum periods which guides in designing and implementing interventions for prevention.

The unclean place of delivery as a risk factor for infection has been supported by another study conducted at Gadchiroli, India, where over 10% of women who delivered at home developed RTIs in the postpartum period (21). Regarding unclean place of delivery, it has been reported by one community-based study in Bangladesh in which observations of trained and practices of untrained dais were made at deliveries. Despite the fact that the trained dai made most safe practices, the prevalence of foul-smelling vaginal discharge remained high in both the groups. This showed that the place of delivery really matters (15). It could be due to unhygienic practices, like the use of unclean sheets and instruments by dais, resulting in chronic pelvic inflammatory diseases followed by secondary infertility. This is quite alarming as national data have shown that 77% of deliveries are being conducted by untrained birth attendants in Pakistan (16).

In our study, non-washing of the perineal area during the postpartum period is significant for secondary infertility. This is not an uncommon practice in South Asia. A study conducted in Dhaka, Bangladesh, suggested that women believed in minimizing their contact with water as this could lead to body aches and disturbed blood flow (22). Similarly, unsafe menstrual practices have been shown by a study conducted in Bangladesh showing that 80% of females re-used the same cloth for absorption of bleeding, and of them, 42% dried the cloth under the sun before re-use. The study further reported that, at times, material was washed, but not sun-dried. The material was dried in a hidden place but, at times, it remained damp which gave rise to microbial growth and insect larvae (12). Another study from Karachi, Pakistan, reported that women at the postpartum period often used vaginal medication. This was either suggested by ‘dai’ or by mothers-in-law. Traditionally, they treat heavy vaginal bleeding and fever with vaginal medication, when women have later reported greenish discharge after treatment (23). This may be due to various factors, such as lack of awareness about the importance of hygienic practices, negligence, or inability to afford the use of clean and hygienic material. Lack of awareness of the use of clean material has been shown by another study that focused on postpartum practices as women presumed the postpartum period as a dirty time period and do not pay attention to the importance of hygiene during this period (22).

It is well-documented that educated women are more employed and those women who are educated and employed have better healthcare-seeking behaviours (24–26). Although the association of low socioeconomic status with high risk for various diseases, including infertility, has been well-demonstrated (27), we did not aim at assessing the association of socioeconomic factors with secondary infertility; hence, we did not include these factors in our final model of analysis. However, there was a higher proportion of controls who were literate and employed compared to cases. This could be indirectly associated with the fact that cases were more likely to be attended by untrained birth attendants that cost less. Moreover, these women being more illiterate are also unaware of hazards of unclean practices during the menstruation and postpartum periods.

There are certain limitations of this study which should be considered before interpreting the results. First, the cases were recruited from selected facilities; thus, generalizibility should be considered with caution. Second, as the information was collected about previous practices, recall bias was a possibility.

However, despite the above-mentioned limitations, the findings of the study are sufficient to propose public-health interventions to address the identified risk factors which are preventable and modifiable to a great extent through properly-focused interventions. There is a strong need for creating awareness among women of hygienic practices during the menstrual, partum and postpartum periods. Moreover, training of birth attendants in the appropriate use of clean sheets and instruments at the time of delivery is mandatory. All pregnant women should be advised to keep clean sheets ready beforehand for their use when required. Also, the instruments to be used by the birth attendants during the partum period should be cleaned and sterilized. Moreover, women need to be counselled on the importance of hygienic practices during the postpartum and menstrual period.
